# Predictive Value of Transabdominal Ultrasonography in Detecting Extrahepatic Bile Duct Obstructive Lesions Compared with Endoscopic Retrograde Cholangiopancreatography

**DOI:** 10.12669/pjms.41.2.9613

**Published:** 2025-02

**Authors:** Abdulkhaleq Ayedh Binnuhaid, Sultan Abdulwadoud Alshoabi, Fahad H. Alhazmi, Awadia Gareeballah, Faisal A. Alrehily, Abdulaziz A. Qurashi

**Affiliations:** 1 Abdulkhaleq Ayedh Binnuhaid, Department of Specialized Surgery, Radiology Section, Faculty of Medicine, Hadhramout University, Hadhramaut, Republic of Yemen; 2 Sultan Abdulwadoud Alshoabi, Department of Diagnostic Radiology, College of Applied Medical Sciences, Taibah University, Al-Madinah Al-Munawwarah, Kingdom of Saudi Arabia; 3 Fahad H. Alhazmi, Department of Diagnostic Radiology, College of Applied Medical Sciences, Taibah University, Al-Madinah Al-Munawwarah, Kingdom of Saudi Arabia; 4 Awadia Gareeballah, Department of Diagnostic Radiology, College of Applied Medical Sciences, Taibah University, Al-Madinah Al-Munawwarah, Kingdom of Saudi Arabia; 5 Faisal A. Alrehily, Department of Diagnostic Radiology, College of Applied Medical Sciences, Taibah University, Al-Madinah Al-Munawwarah, Kingdom of Saudi Arabia; 6 Abdulaziz A. Qurashi, Department of Diagnostic Radiology, College of Applied Medical Sciences, Taibah University, Al-Madinah Al-Munawwarah, Kingdom of Saudi Arabia

**Keywords:** Obstructive jaundice, Transabdominal ultrasonography (TAUS), Endoscopic retrograde cholangiopancreatography (ERCP), Choledocholithiasis, Cholangiocarcinoma, Pancreatic carcinoma, Bile duct stricture, External compression

## Abstract

**Background & Objective:**

Transabdominal ultrasonography (TAUS) remains the initial imaging modality in diagnosis of bile duct obstructive lesions. The purpose of this study was to investigate the predictive value of TAUS in detecting bile duct obstructive lesions in comparison with endoscopic retrograde cholangiopancreatography (ERCP) as the standard method.

**Methods:**

This retrospective descriptive study analyzed the electronic records of the patients diagnosed with obstructive jaundice from April 2017 to November 2022 at Alsafwa Consultative Medical Center in Almukalla City, Hadhramout, Yemen. All patients involved were diagnosed by TAUS and the diagnosis was confirmed by ERCP. A comparison of the diagnoses was performed.

**Results:**

TAUS and ERCP demonstrated high compatibility in bile duct obstructive lesions, with substantial agreement in detecting cholangiocarcinoma, bile duct stricture, stones, pancreatic cancer, and ampulla of Vater mass (compatibility ranging from 71.4% to 100%, Cohen’s Kappa = 0.748, *p* < 0.001). Pearson correlation indicated strong agreement between the two methods (r=0.856). TAUS showed high sensitivity, and positive predictive value (PPV), particularly for bile duct stones (99.4% sensitivity, 86.7% PPV), and pancreatic carcinoma (94.3% sensitivity, 82.5% PPV), with significant effectiveness in identifying other conditions like bile duct stricture (42.6% sensitivity, 88.5% PPV) and cholangiocarcinoma (70.6% sensitivity, 100% PPV). (*p*<0.001). Overall, TAUS and ERCP displayed excellent compatibility across various diagnoses, with near-perfect agreement in determining the causes in the ampulla of Vater and bile duct (Kappa= 0.899, p<0.001)

**Conclusion:**

TAUS is a reliable and highly valuable imaging modality for detecting and determining the cause and level of bile duct obstruction in patients with obstructive jaundice which offers a non-invasive approach, radiation free, with minimal risk of serious complications.

## INTRODUCTION

Accurate preoperative diagnosis of common bile duct (CBD) obstructive lesions is essential for optimizing surgical planning and minimizing complications. In many institutions, transabdominal ultrasonography (TAUS) is often considered as the initial imaging modality employed to identify abnormalities in the CBD.^1^ Ultrasonography offers a non-invasive, radiation-free, cost-effective approach, with the added real-time evaluation and high spatial resolution. These features make it particularly useful for detection and differential diagnosis of gallbladder lesions.^2^ However, it is important to note that ultrasonography is an operator dependent, that is requiring a high level of expertise and skills. The quality of ultrasound image is influenced by various factors, including probe selection, patient position, and machine setting. Additionally, operators must be aware of common artifacts in the hepatobiliary system imaging, such as acoustic shadowing artifact, reverberation artifact, and ring down artifact, to avoid misdiagnosis these as CBD lesions.^3^

Endoscopic retrograde cholangiopancreatography (ERCP) is an established diagnostic and therapeutic tool for lesions of the biliary tree and the pancreas. It combines endoscopic and radiologic imaging, but its reliance on fluoroscopy exposes endoscopists to radiation risk.^4^ Due to its invasive nature, ERCP is typically not the first choice for investigating CBD issues; non-invasive imaging modalities such as magnetic resonance cholangiopancreatography (MRCP) are usually preferred.^5^ ERCP’s potential complications include acute pancreatitis, post-sphincterotomy bleeding, infections, cardiopulmonary complications, and perforations, necessitating its use only under strong indications.^6^

In January 2010, the American Society for Gastrointestinal Endoscopy (ASGE) issued consensus guidelines for managing patients with suspected choledocholithiasis.^7^ These guidelines categorize patients with symptomatic cholelithiasis into high, intermediate, or low risk for choledocholithiasis based on clinical, laboratory, and ultrasonography criteria. Patients at high risk (>50% probability of CBD stone) are recommended to undergo ERCP directly. Those at intermediate risk (10-50% probability of CBD stone) should be evaluated further with endoscopic ultrasonography (EUS), MRCP, Laparoscopic ultrasonography, or intraoperative cholangiography (IOC). Patients at low risk (<10% probability of CBD stone) should proceed directly to cholecystectomy without the need for ERCP or additional imaging.^7^ In Asian society, in selective high-risk cases, EUS is still be suggested as the first-line approach, potentially - avoiding ERCP in patients with negative findings for CBD stones on EUS.^8^

There is a notable scarcity in the literature regarding the sensitivity and specificity of TAUS in detecting of CBD stones. This study aimed to address this gap of knowledge by investigating the diagnostic accuracy of TAUS for definitive diagnosis in patients with obstructive lesions in the extrahepatic bile ducts. Such an investigation is particularly pertinent in developing countries, where TAUS remains a widely available imaging modality preceding the use of ERCP.

## METHODS

This retrospective cross-sectional study analyzed the electronic patients records documenting cases of bile duct obstruction from April 2017 to November 2022 at Alsafwa Consultative Medical Center (ACMC) in Almukalla city, Hadhramout, Republic of Yemen.

### Institutional review board statement:

This study was approved by the Research Ethics Committee of Alsafwa Consultative Medical Center (ACMC) in Almukalla city, Hadramout, Republic of Yemen (Approval Number: ACMC-12-23). All procedures were carried out in conformity with the Declaration of Helsinki and all applicable standards and laws.

### Informed consent statement:

Patient’s consents were waived due to the retrospective nature of the study.

### Sample size:

In this study, 295 patients with clinically diagnosed obstructive jaundice were evaluated using TAUS and ERCP to identify the cause of bile duct obstruction. The study excluded patients whose bile duct obstruction was diagnosed by TAUS but not confirmed by ERCP, and those whose obstruction causes were not conclusively determined by ERCP.

### Data acquisition:

All patients underwent TAUS, performed by a highly qualified radiologist with 13 years postdoctoral experience in ultrasound imaging. A 3.5 MHz deep curved transducer on a Mindray DC30 ultrasound Machine was used to assess the CBD and biliary ducts in all patients. Subsequently, each patient also underwent ERCP to confirm the diagnoses of bile duct obstruction.

### Ultrasound imaging procedure:

**Fig.1:**
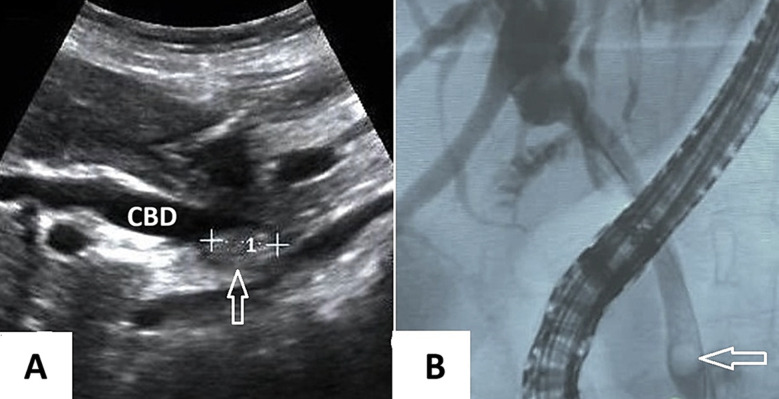
A) A Transabdominal Ultrasonography image showing dilatation of the common bile duct (CBD) with a large stone (arrow) impacted near the distal part of it. B) A cholangiography image showing dilatation of the CBD and biliary ducts with a filling defect (arrow) at the distal end, which proved to be a CBD stone.

After fasting for at least six hours, patients underwent TAUS scanning in both supine and where necessary, left lateral decubitus positions to effectively scan the biliary tree. The ultrasound probe, after applying a contact gel, was placed in the right subcostal region. TAUS scanning of the right upper quadrant region was performed in the sagittal, coronal, and axial planes. From our work, we select an ultrasonography image, and a radiographic image of ERCP to demonstrate the difference between the two images (Fig.1).

### ERCP technique:

Following the revised guidelines of the ASGE as reported by Jacob et al.,^9^ each patient in this study was underwent ERCP for both diagnostic and therapeutic purposes. The perioperative managements of patients adhered to the consensus guidelines reported by Azimaraghi et al.^10^

### Statistical analysis:

The collected data were analyzed using the Statistical Package for Social Sciences (SPSS) version 25 (IBM, Armonk, NY). DATAtab Team (2023), (DATAtab: Online Statistics Calculator (DATAtab e. U. Graz, Austria) was used to visualize the findings. Fleiss’ kappa measurement of agreement was used to measure compatibility between TAUS and ERCP. Additionally, the Jamovi software medical decision calculator (The jamovi project (2023). *Jamovi*. (Version 2.4) [Computer Software]. Retrieved from https://www.jamovi.org.) was used to calculate the sensitivity and positive predictive value (PPV) of ultrasonography in diagnosing various pathologies of the biliary system, using ERCP as the gold standard method. We reported on the common causes and anatomical locations of the bile duct obstruction in the extrahepatic bile ducts. The reported causes and locations of obstruction identified by the TAUS and the ERCP were compared. The agreement between the findings from TAUS and ERCP was assessed using the Kappa statistic (Cohen’s Kappa), with Kappa values < 0 indicating no agreement; Kappa 0.01-0.20 indicating no to slight agreement; Kappa 0.21-0.40 indicating fair agreement; Kapa 0.41-0.60 indicating moderate agreement; Kappa 0.61-0.80 indicating substantial agreement, and Kappa 0.81-1 indicating almost perfect agreement.^11^

## RESULTS

### Demographic data:

This study involved 295 patients, aged 57±18.13 (range: 18-95 years), of whom 60.70% (n=179) were females and 39.30% (n=116) were males.

### Causes of obstruction by TAUS and by ERCP:

TAUS revealed that bile duct stones were the most common cause of bile duct obstruction (n=203, 68.8%), followed by carcinoma of the pancreas (n=40, 13.6%), bile duct stricture (n=26, 8.8%), ampulla of Vater mass (n=14, 4.7%), and cholangiocarcinoma (n=12, 4.1%), while ERCP revealed that bile stones were the most common cause of bile duct obstruction (n=177, 60%), followed by bile duct stricture (n=54, 18.3%), carcinoma of the pancreas (n=35, 11.9%), cholangiocarcinoma (n=17, 5.8%), and ampulla of Vater mass (n=12, 4.1%) (Table 1).

**Table-I:** Cause of bile duct obstruction according to Transabdominal Ultrasonography (TAUS) and Endoscopic Retrograde Cholangiopancreatography (ERCP).

Causes of bile duct obstruction	Cause according to TAUS	Cause according to ERCP

	Number (%)	Number (%)
Bile duct stone	203 (68.8%)	177 (60%)
Bile duct stricture	26 (8.8%)	54 (18.3%)
Cholangiocarcinoma	12 (4.1%)	17 (5.8%)
Carcinoma of the pancreas	40 (13.6%)	35 (11.9%)
Ampulla of Vater mass	14 (4.7%)	12 (4.1%)
Total	295 (100%)	295 (100%)

### Compatibility in detecting causes of bile duct obstruction:

Cross tabulation between the causes of bile duct obstruction was determined by TAUS and ERCP that showed high compatibility: 100% in identifying Cholangiocarcinoma, 88.5% for bile duct stricture, 86.7% for bile duct stone, 82.5% for pancreatic cancer, and 71.4% for ampulla of Vater mass. This was substantiated by a substantial agreement on the Cohen’s Kappa (Kappa= 0.748, *X*^2^=711.14, and p<0.001) (Table-II). Additionally, Pearson correlation indicated strong compatibility between the diagnoses of TAUS and ERCP (r=0.856).

**Table-II:** Cross tabulation test between the cause of bile duct obstruction according to ultrasonography (rows), and Endoscopic Retrograde Cholangiopancreatography (columns).

Causes of bile duct obstruction	Bile duct stone	Bile duct stricture	Cholangiocarcinoma	Carcinoma of the pancreas	Ampulla of Vater mass	Total

	No. (%)	No. (%)	No. (%)	No. (%)	No. (%)	
Bile duct stone	176 (86.7%)	25 (12.3%)	1 (0.5%)	1 (0.5%)	0 (0.0%)	203
Bile duct stricture	0 (0.0%)	23 (88.5%)	2 (7.7%)	0 (0.0%)	1 (3.8%)	26
Cholangiocarcinoma	0 (0.0%)	0 (0.0%)	12 (100%)	0 (0.0%)	0 (0.0%)	12
Pancreatic carcinoma	0 (0.0%)	5 (12.5%)	1 (2.5%)	33 (82.5%)	1 (2.5%)	33
Ampulla of Vater mass	1 (7.1%)	1 (7.1%)	1 (7.1%)	1 (7.1%)	10 (71.4%)	14
Total	177	54	17	35	12	295 (100%)

The measurement of agreement (Kappa= 0.748, X^2^=711.14, and p<0.001).

**Fig.2:**
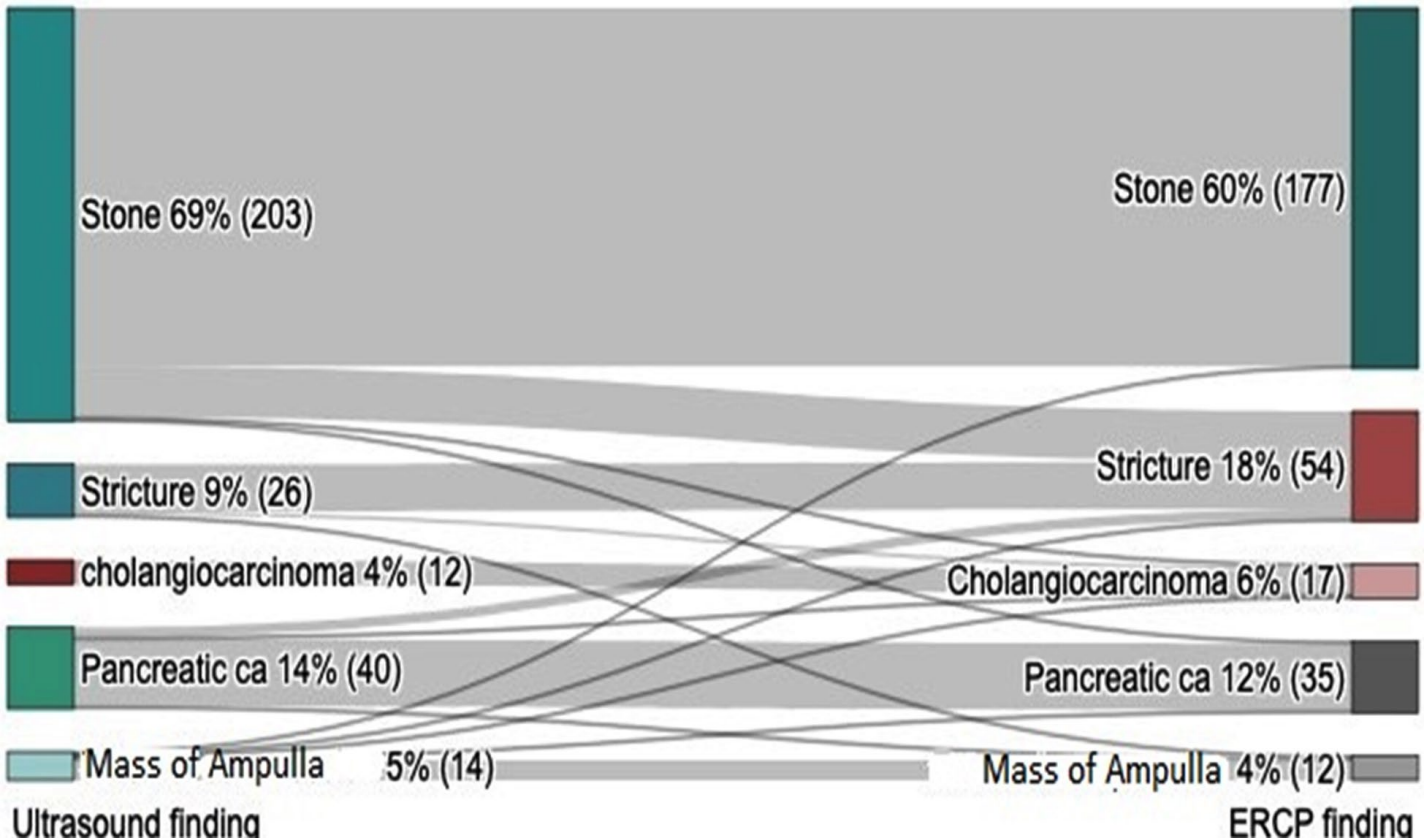
shows the cross-tabulation between the causes of bile duct obstruction determined by TAUS and ERCP (Kappa= 0.748, and p<0.001).

A Sankey diagram further elucidated these findings (Fig.2). For instance, of 177 cases of stone, TAUS correctly identified 176, with one case falsely identified as ampulla of Vater mass. Of the 54 bile duct strictures, 23 were correctly identified by TAUS, while 25 were misidentified as bile duct stones, five as pancreatic carcinoma, and one as ampulla of Vater mass. Similarly, TAUS accurately identified 12 out of 17 cholangiocarcinoma cases, with misidentifications including 2 as bile duct strictures, one as a bile duct stone, one pancreatic cancer, and one as an ampulla of Vater mass. In pancreatic carcinoma cases (n=35), TAUS was accurate in 33 instances, with 1 misidentified as a bile duct stone and another as ampulla of Vater mass. Lastly, of the 12 ampullas of Vater mass cases, TAUS accurately identified 10, with 1 misidentified as a bile duct stricture and another as pancreatic carcinoma.

Fleiss’ kappa measurement of agreement between TAUS and ERCP indicated a high level of diagnostic agreement. There was perfect agreement in diagnoses of pancreatic carcinoma (Kappa=0.863, 95%CI 0.749-0.977) and cholangiocarcinoma (Kappa=0.819, 95%CI 0.705-0.933), substantial agreement for bile ducts stone (Kappa=0.793, 95%CI 0.679-0.907), and ampulla of Vater mass (Kappa=0.759, 95%CI 0.645-0.873), and moderate agreement for bile duct stricture (Kappa=0.508, 95%CI 0.394-0.622). The overall agreement between the two diagnostic methods was substantial (Kappa=0.746, 95%CI 0.675-0.817) (Table-III).

**Table-III:** Fleiss’ kappa measurement of agreement between TAUS and ERCP in detecting the causes of bile duct obstruction.

Ratings			95% CI	

	Fleiss’ kappa	SE	Lower	Upper
Overall	0.748	0.036	0.675	0.817
Stone	0.793	0.058	0.679	0.907
Stricture	0.508	0.058	0.394	0.622
Cholangiocarcinoma	0.819	0.058	0.705	0.933
Pancreatic carcinoma	0.863	0.058	0.749	0.977
Mass of the Ampulla	0.759	0.058	0.645	0.873

***Note:*** 295 subjects/items and 2 methods/measurements.

Our study demonstrated that TAUS is an effective imaging modality for diagnosing bile duct lesions, exhibiting high predictive value across various conditions. For bile duct stones, TAUS showed a sensitivity of 99.4% and a PPV of 86.7%. In cases of pancreatic carcinoma, the sensitivity was 94.3% and PPV was 82.5%. For bile duct strictures, TAUS had a sensitivity of 42.6% and a PPV of 88.5%. Cholangiocarcinoma was detected with a sensitivity of 70.6% and a PPV of 100%. For ampulla of Vater mass as a cause of obstruction, the sensitivity was 83.3% and PPV was 71.4% (*p*<0.001) (Table-IV).

**Table-IV:** Shows sensitivity, and PPV of TAUS in diagnosis of bile duct obstructive lesions taking ERCP as the standard.

Variable	Categories	Values	95% confidence interval		p-value

			Lower	Upper	
Bile duct stone	Sensitivity	99.4 %	96.9 %	100.0 %	<0.001
	PPV	86.7 %	81.2 %	91.0 %	
Bile duct stricture	Sensitivity	42.6 %	29.2 %	56.8 %	<0.001
	PPV	88.5 %	69.8 %	97.6 %	
Cholangiocarcinoma	Sensitivity	70.6 %	44.0 %	89.7 %	<0.001
	PPV	100.0 %	73.5 %	100.0 %	
Pancreas carcinoma	Sensitivity	94.3 %	80.8 %	99.3 %	<0.001
	PPV	82.5 %	67.2 %	92.7 %	
Mass of the Ampulla	Sensitivity	83.3 %	51.6 %	97.9 %	<0.001
	PPV	71.4 %	41.9 %	91.6 %	

ERCP= Endoscopic Retrograde Cholangiopancreatography, PPV= Positive predictive value, CI= Confidence interval.

### Compatibility in determining locations of bile duct obstruction:

Cross-tabulation test between TAUS and ERCP showed a high degree of compatibility in determining causes of bile duct obstruction. Specifically, there was 100% compatibility in identifying lesions in the ampulla of Vater, 99.6% in the bile duct, 83.3% for external compression by liver masses, and 81.6% for pancreatic causes. This high level of agreement was further substantiated by a perfect score on Cohen’s Kappa (Kappa= 0.899, *X*^2^=685.99, *p*<0.001) as presented in Table-V. Additionally, the Pearson correlation analysis revealed strong compatibility (r=0.97) between the locations determined by TAUS and ERCP.

**Table-V:** Cross tabulation test between the anatomical locations of bile duct obstruction according to Transabdominal Ultrasonography (rows), and Endoscopic Retrograde Cholangiopancreatography (columns).

	Endoscopic Retrograde Cholangiopancreatography (ERCP)					

	Parameters	Common bile duct	Head of the pancreas	Ampulla of vater	Compression by liver mass	Total
Transabdominal Ultrasonography (TAUS)	Common bile duct	240 (99.6%)	0 (0.0%)	1 (0.4%)	0 (0.0%)	241
	Head of the pancreas	5 (13.2%)	31 (81.6%)	1 (2.6%)	1 (2.6%)	38
	Ampulla of Vater	0 (0.0%)	0 (0.0%)	10 (100%)	0 (0.0%)	10
	Compression by liver mass	1 (16.7%)	0 (0.0%)	0 (0.0%)	5 (83.3%)	6
	Total	246 (83.4%)	31 (10.5%)	12 (4.1%)	6 (2%)	295 (100%)

The measurement of agreement (Kappa= 0.899, X^2^=685.99, p<0.001).

**Fig.3:**
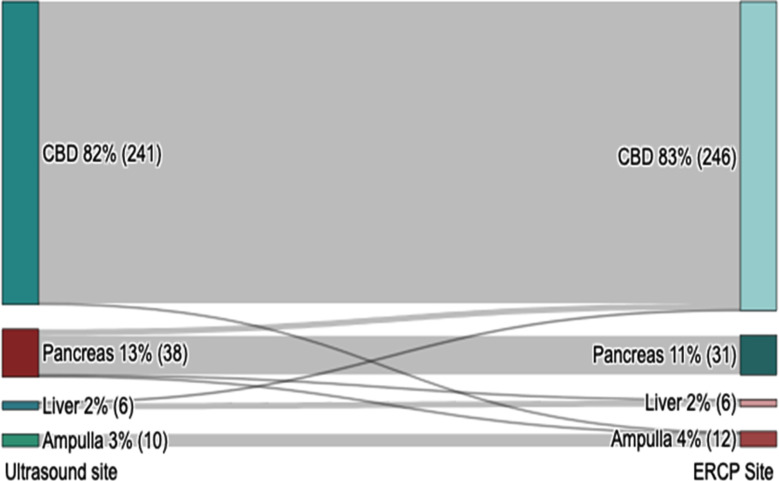
Shows the cross-tabulation between the sites of bile duct obstructive lesions determined by TAUS and ERCP (Kappa=0.899, p<0.001).

The detailed findings, illustrated in a Sankey diagram (Fig.3) further reinforce the accuracy of TAUS. Out of 246 cases involving the common bile duct (CBD), TAUS correctly identified 240, with five cases incorrectly marked as pancreatic cancers, and only one case incorrectly marked as external compression by a liver mass. It accurately identified all 31 pancreatic lesions. In the Ampulla of Vater, TAUS correctly identified 10 out of 12 lesions, with the remaining one each being misclassified as CBD and pancreatic lesions. Finally, in the cases of liver lesions causing external compression, TAUS accurately identified five out of six cases, and misclassified only one as a pancreatic lesion (Kappa=0.899, *X*^2^=685.99, *p*<0.001).

Additionally, Fleiss’ kappa measurement of agreement between TAUS and ERCP shows perfect agreement in determining the locations of the lesions causing bile duct obstruction (Kappa=0.899, 95%CI 0.812-0.987) (Table-VI).

**Table-VI:** Fleiss’ kappa measurement of agreement between TAUS and ERCP in determining locations of the causes of bile duct obstruction.

Ratings	Fleiss’ kappa	SE	95% CI	

			Lower	Upper
Overall	0.899	0.045	0.812	0.987
CBD	0.918	0.058	0.804	1.032
Pancreas	0.885	0.058	0.771	0.999
Ampulla	0.906	0.058	0.792	1.020
Liver	0.830	0.058	0.716	0.944

***Note:*** 295 subjects/items and 2 methods/measurements.

Our study demonstrated that TAUS is an effective imaging modality for determining locations of bile duct lesions, exhibiting high diagnostic accuracy across various locations. For bile duct stones, TAUS showed a sensitivity of 97.6% and a PPV of 99.6%. In determining cases of pancreatic lesions, the sensitivity was 100% and PPV was 81.6%. For determining ampulla of Vater lesions, TAUS had a sensitivity of 83.3% and a PPV of 100%. Finally, TAUS was identifying liver masses compressing bile ducts with a sensitivity of 83.3% and a PPV of 83.3% (*p*<0.001) (Table-VII).

**Table-VII:** Shows sensitivity, and PPV of TAUS in determining locations of bile duct obstructive lesions taking ERCP as the standard.

Variable	Categories	Values	95% confidence interval		p-value

			Lower	Upper	
CBD	Sensitivity	97.6 %	94.8 %	99.1 %	<0.001
	PPV	99.6 %	97.7 %	100.0 %	
Pancreas	Sensitivity	100 %	88.8 %	100.0 %	<0.001
	PPV	81.6 %	65.7 %	92.3 %	
Mass of the Ampulla	Sensitivity	83.3 %	51.6 %	97.9 %	<0.001
	PPV	100 %	69.2 %	100.0 %	
Liver mass	Sensitivity	83.3 %	35.9 %	99.6 %	<0.001
	PPV	83.3 %	35.9 %	99.6 %	

ERCP= Endoscopic Retrograde Cholangiopancreatography, PPV= Positive predictive value, CI= Confidence interval.

## DISCUSSION

Optimal diagnosis of obstructive lesions in the CBD is crucial for correct treatment planning. In resource-limited settings, TAUS serves as the initial imaging modality in preliminary diagnostic stratification and management planning of patients with bile duct obstructive lesions. This study aimed to evaluate the diagnostic accuracy of TAUS in detecting CBD stones and other obstructive lesions prior to undergoing diagnostic and therapeutic ERCP. Accurate visualization through ERCP is essential before definitive surgery, as CBD stones are a common cause of obstructive jaundice.^12^ This study, while primarily assessing the predictive value of TAUS, also acknowledges the role of ERCP not only as a diagnostic gold standard for CBD stones but also as a preferred treatment approach, often involving endoscopic biliary sphincterotomy or endoscopic papillary balloon dilation, for these obstructive conditions as reported by Hu et al.^13^

Our study demonstrated a substantial agreement between TAUS and ERCP in detecting CBD obstructive lesions. We observed 99.4% sensitivity and an 86.7% PPV for TAUS in identifying bile duct stones. These results align with Alkarboly et al., who reported a sensitivity of 80% and a PPV of 95.65% for TAUS in diagnosing bile duct stones.^14^ However, our findings are in contrast with those of another study by Samanta et al, which found a sensitivity of 49.12%, and a PPV of 98.25% for TAUS, compared with the intra-operative findings.^15^ Additionally, Hu et al. noted an increase in the sensitivity and specificity of TAUS for screening bile duct stones with advancing age.^13^

In our study, we observed a high degree of compatibility between TAUS and ERCP in in diagnosing various conditions, with 100% compatibility in determining Cholangiocarcinoma, 88.5% for bile duct stricture, 86.7% for bile duct stone, 82.5% for pancreatic cancer, and 71.4% for ampulla of Vater mass, demonstrating substantial agreement (Kappa= 0.748, p<0.001). Particularly for CBD stones, our findings align with those of De Silva et al., who also reported strong compatibility between TAUS and ERCP in detecting, quantifying, and localizing these stones.^16^ This agreement reinforces the value of TAUS as a primary, non-invasive imaging modality. Its accessibility and safety profile, free from serious complications or radiation hazards, make it an especially advantageous tool in initial diagnostic assessments.

Our results demonstrated varying levels of sensitivity for TAUS: 99.4% for bile duct stones, 94.3% for pancreatic carcinoma, 83.3% for ampulla of Vater mass, 70.6% for cholangiocarcinoma, and 40.4% for bile duct stricture. These findings are in line with those of Zahur et al., who reported 76.2%, 81.3%, and 76.8% sensitivity, specificity, and diagnostic accuracy for TAUS in detecting CBD stones.^17^ Fadahunsi et al. also noted high sensitivity of TAUS in diagnosing cholangiocarcinoma (100%), pancreatic carcinoma (81%), and bile duct stones (70.7%), with an overall sensitivity of 76.6%.^18^

However, our study revealed a lower accuracy (42.6%) in diagnosing of bile duct stricture. This aligns with findings by Singh et al. and Dumonceau et al., who noted that bile duct strictures often present diagnostic challenges due to their diverse etiologies, which can be inflammatory, infectious, iatrogenic, ischemic, or malignant. These strictures are difficult to definitively diagnose with imaging alone, and sometimes even with ERCP and biliary sampling.^19^,^20^

Our results also showed a high compatibility between TAUS and ERCP in determining the location of bile duct obstruction (Kappa=0.899, p<0.001, 95%CI 0.812-0.987), with the distal part being the most common site of obstructive lesions. This is consistent with the findings of Fadahunsi et al., who reported the intra-pancreatic CBD as the frequent site of obstruction.^18^ This trend may be explained by the common impaction of CBD stones at the distal part^21^, and the fact that two-thirds of pancreatic carcinoma develop in the pancreas head, often presenting with progressive obstructive jaundice^22^, and all lesions of the ampulla of Vater are intuitively at the distal end of the CBD.

Finally, our results demonstrated high levels of sensitivity and predictive value for TAUS for determining levels and locations of obstructive lesions: 97.6% and 99.6% for CBD, 83.3% and 100% for ampulla of Vater mass, 100 and 81.6% for pancreas, and 71.4% for compressing liver masses. This trend may be explained by that CBD are the commonest cause of biliary obstruction.^23^ Additionally, TAUS examining in various techniques has a potential impact on detecting pancreatic cancer with good accuracy.^24^

### Strength of the study:

The strength of this study is that it investigated the accuracy of a widely available non-invasive imaging modality using high sample size of confirmed causes of obstructive jaundice which is a common health problem in daily clinical practice. The study adds the common causes of obstructive jaundice and the diagnostic accuracy of TAUS which is the available imaging modality for this problem in Hadhramout region.

### Limitations:

One limitation of this study is the inherent challenge with TAUS in precisely determining the locations of the etiologies causing obstructive jaundice within different segments of the CBD. This lack of specific locational data was particularly notable when comparing the locations of CBD stones.

## CONCLUSION

This study concluded that TAUS is a reliable and highly valuable imaging modality for initially diagnosing the cause and level of bile duct obstruction in obstructive jaundice patients. TAUS demonstrated high predictive value in identifying bile duct stones, pancreatic carcinoma, external compression, and cholangiocarcinoma, and it maintains acceptable predictive value in determining bile duct stricture. Its advantages include easy accessibility, non-invasive, radiation free, and minimal risk of serious complications.

### Data availability statement:

Data are available from the corresponding author up on a reasonable request.

### Author`s Contribution:

**SAA** formed the study concept, performed the data analysis and prepared the manuscript.

**AAB** provided ultrasound examinations, data collection and interpretation.

**FHA and AG** revised data analysis.

**FAA** revised the manuscript and edited language.

**AAQ** revised the final manuscript.

All authors have read and agreed to the final version of the manuscript and are responsible for the integrity of the contents of the article.
